# Towards Positional Isolation of Three Quantitative Trait Loci Conferring Resistance to Powdery Mildew in Two Spanish Barley Landraces

**DOI:** 10.1371/journal.pone.0067336

**Published:** 2013-06-24

**Authors:** Cristina Silvar, Dragan Perovic, Thomas Nussbaumer, Manuel Spannagl, Björn Usadel, Ana Casas, Ernesto Igartua, Frank Ordon

**Affiliations:** 1 Department of Ecology, Plant and Animal Biology, University of Coruña, A Coruña, Spain; 2 Institute for Resistance Research and Stress Tolerance, Julius Kühn-Institute, Quedlinburg, Germany; 3 MIPS/IBIS, Helmholtz Zentrum München, Neuherberg, Germany; 4 Institute for Biology I, RWTH Aachen University, Aachen, Germany; 5 Department of Genetics and Plant Production, Aula Dei Experimental Station (Consejo Superior de Investigaciones Científicas), Zaragoza, Spain; New Mexico State University, United States of America

## Abstract

Three quantitative trait loci (QTL) conferring broad spectrum resistance to powdery mildew, caused by the fungus *Blumeria graminis* f. sp. *hordei*, were previously identified on chromosomes 7HS, 7HL and 6HL in the Spanish barley landrace-derived lines SBCC097 and SBCC145. In the present work, a genome-wide putative linear gene index of barley (Genome Zipper) and the first draft of the physical, genetic and functional sequence of the barley genome were used to go one step further in the shortening and explicit demarcation on the barley genome of these regions conferring resistance to powdery mildew as well as in the identification of candidate genes. First, a comparative analysis of the target regions to the barley Genome Zippers of chromosomes 7H and 6H allowed the development of 25 new gene-based molecular markers, which slightly better delimit the QTL intervals. These new markers provided the framework for anchoring of genetic and physical maps, figuring out the outline of the barley genome at the target regions in SBCC097 and SBCC145. The outermost flanking markers of QTLs on 7HS, 7HL and 6HL defined a physical area of 4 Mb, 3.7 Mb and 3.2 Mb, respectively. In total, 21, 10 and 16 genes on 7HS, 7HL and 6HL, respectively, could be interpreted as potential candidates to explain the resistance to powdery mildew, as they encode proteins of related functions with respect to the known pathogen defense-related processes. The majority of these were annotated as belonging to the NBS-LRR class or protein kinase family.

## Introduction

Barley (*Hordeum vulgare* L.) was domesticated in the Fertile Crescent about 10,000 years ago [1], [2] and nowadays it ranks as the fourth cereal in worldwide production after wheat, rice and maize [3]. As for other crops, domestication and modern plant breeding have endangered the maintenance of the genetic diversity of barley [4], [5]. Although, overall, barley genetic diversity has not decreased [6] local dominance of cultivars poses a serious threat to sustainable production, especially considering the risks associated to the appearance of new strains of pathogens that may be virulent on all cultivars grown in a region [7], [8]. The limited genetic variation regarding disease tolerance is a great concern as it may result in widespread crop-yield and quality losses if new virulent pathogen populations appear [9]. Incorporation of new genes or alleles that confer pathogen resistance is needed to alleviate this genetic vulnerability. Wild relatives and landraces probably represent the most valuable reservoirs of unexploited variability within the primary gene pool of barley. For this reason, they have had, and still have, enormous relevance in breeding for disease resistance [10]–[13].

The Spanish Barley Core Collection (SBCC) [14] is constituted by a representative sample of the landraces cultivated in Spain before the advent of modern breeding. It consists of inbred lines derived from native landraces with an important history of adaptation and selection under Mediterranean conditions [15], [16]. In order to evaluate its potential to contribute new genetic diversity to enhance the disease resistance of barley, the SBCC was screened with several fungal and viral pathogens. High levels of broad resistance to the fungus *Blumeria graminis* f.sp. *hordei,* responsible for powdery mildew, were detected in some lines tested with a large variety of isolates [17], [18]. The resistances from the two most interesting lines were investigated further in mapping populations resulting in the identification of different sets of quantitative trait loci (QTL). Two QTL were identified on the chromosome 7H in the Spanish barley landrace-derived line SBCC097 [19]. In a recent work, the chromosomal intervals containing these resistances have been subjected to marker saturation following a comparative genomic approach based on the synteny of barley with the reference genomes of rice, sorghum and Brachypodium [20]. In a second line, i.e. SBCC145, a major QTL with a large effect was located to the long arm of chromosome 6H accounting for ca. 60% of the phenotypic variance [21]. The position and magnitude of effects of these QTLs, an exhaustive analysis based on a set of *B. graminis* pathotypes with broad spectra of virulences [18], and the characteristics of the defense reaction at the cellular level [22] suggested that they are newly identified loci or alleles for non-race specific resistance against powdery mildew in cultivated barley. The effective use of these resistance genes in barley breeding, avoiding linkage drag, requires a precise localization or, even better, the identification of candidate genes. Such identification of candidates is essential to ascertain the biochemical and physiological mechanisms underlying the resistances.

The progress towards map-based cloning of these QTLs and their exploitation in barley breeding programs follows a series of steps: first positioning these resistance loci on the barley genome through the development of closely linked markers, then identifying putative candidate genes together with aid of high resolution mapping populations. In this regard, recent advances in barley genomics enable researchers to go one step further in the shortening and explicit demarcation on the barley genome of the regions conferring resistance to powdery mildew as well as in the identification of candidate genes. Mayer et al. [23], [24] developed the first Genome Zipper of a Triticeae genome, comprising a putative linear gene index of each chromosome in barley, embedded in a comparative grass genome organization model. The barley Genome Zipper led to the assignment of 86% of the estimated barley genes to individual chromosome arms and their organization in a virtual gene map. More recently, the International Barley Genome Sequencing Consortium (IBSC) published a cumulative physical map of 4.98 Gb and a draft of the barley genomic sequence holding 26,159 “high-confidence” genes [25].

In the present work, we took advantage of all these genomic resources to go ahead on our attempt to genetically dissect and physically circumscribe the barley regions specifically conferring resistance to powdery mildew in the Spanish lines SBCC097 and SBCC145.

## Materials and Methods

### Plant and Pathogen Materials

The SBCC097×Plaisant F_5_ and F_6_ RIL population (262 lines) was used to select 13 lines as the most informative ones for their clear-cut phenotypic responses and the unequivocal presence of just one of the two QTL on 7H, based on marker information obtained previously [20]. The doubled haploid (DH) population SBCC145×Beatrix, originally with over 400 lines, was employed for the selection of 13 DH lines showing recombination between flanking markers 11_1351 and 11_0509 on the chromosome 6HL [21].

Phenotypic disease scores of these lines against four *B. graminis* isolates (R79, R180, R126 and R178) used to screen the SBCC097×Plaisant RIL population and two isolates (R211 and R224) employed to inoculate the SBCC145×Beatrix DH population were available from earlier works [17], [18].

### Comparative Analysis to the Barley Genome Zipper

The closest markers flanking the QTLs identified on chromosomes 6H and 7H in previous works, were employed to select the target region for comparison to the barley Genome Zipper developed by Mayer et al. [24]. Data available at the MIPS/IBIS (http://mips.helmholtz-muenchen.de/plant/barley/gz/index.jsp) were used. The sequences of rice, sorghum and Brachypodium genes located at the promising regions on the barley Genome Zipper were downloaded from the *Oryza sativa* ssp. *japonica* IRGSP Build5 (http://www.rapdb.dna.affrc.go.jp), *Sorghum bicolor* release v1.0 (http://www.phytozome.net/sorghum.php) and *Brachypodium distachyon* JGI 8× (http://www.brachypodium.org). The sequences of those genes were used as queries for BlastN search at the ViroBlast tool implemented for barley at the Leibniz-Institute of Plant Genetics and Crop Plant Research (http://webblast.ipk-gatersleben.de/barley/viroblast.php). ViroBlast was performed with cut-off parameters of E-value ≤e^−10^, identity, ≥80% and a minimum of 100 bp match length against the database “assembly_WGSMorex”, which holds contigs information on sequences from the cv. Morex at coverage of ∼50×. The contigs with the best hit were employed in a second step of ViroBlast against the “sorted Chromosomes” database, harbouring 454 reads of flow sorted chromosomes of cv. Betzes. In those cases in which no reference gene (rice, sorghum or Brachypodium) was available, the “reads matching marker stringent” obtained from Mayer et al. [24] were directly employed for marker development.

### Marker Development and Genotyping

With the aim of developing new molecular markers derived from Morex contigs at the target chromosomal regions, the 454 reads of Betzes, identified in the second step of BlastN, were aligned with their respective Morex contigs using the software Sequencher™ version 4.5 (Genes Codes Corporation, Ann Arbor, MI, USA). *In silico* SNPs were identified between cvs. Morex and Betzes and primers flanking those SNPs were designed using the software BatchPrimer3 v1.0 [26].

Routine PCR was done in 20 µl reaction volume including 25–50 ng genomic DNA, 0.5 U of Taq DNA Polymerase (Solis Biodyne, Tartu, Estonia) 1× PCR reaction buffer, 1.5 mM MgCl2, 0.2 mM dNTPs and 0.2 µM of each primer. All fragments were amplified using a previously published touchdown PCR profile [20]. Purified amplicons were subjected to cycle-sequencing from both ends on the ABI377XL sequencer using BigDye v3.1 terminator sequencing chemistry (ABI Perkin Elmer, Weiterstadt, Germany). Sequence analysis and identification of polymorphisms were conducted using the Sequencher™ software. The SNPs between the parental lines were transformed to CAPS (Cleaved Amplified Polymorphism) markers. Restriction digestion was performed as described earlier [20]. Markers in which polymorphism was detected between the parental lines, either length or presence/absence, were genotyped directly. The markers developed were named after the corresponding Morex contig name (assembly_WGSMorex, ∼55×, http://webblast.ipk-gatersleben.de/barley/docs/blast_databases.html) preceded by the prefix QB (Quedlinburg Barley).

### Linkage and QTL Analysis

All informative lines for the two populations, i.e. lines that showed recombination across the target intervals, were used to map the new markers. The previously mapped BOPA or microsatellite markers on 6H and 7H [19], [21] were employed as a framework to place the new markers. Genetic distances were calculated by minimizing the number of recombinants within the progeny. Linkage analyses were performed with JoinMap 4.0 [27], using Kosambi’s map function and a minimum logarithm of the odds ratio (LOD score) of 3. QTL analysis was performed using the Multiple QTL Model (MQM) [28] implemented in MapQTL 5.0 [29]. Several rounds of analysis with cofactors were conducted until a stable LOD profile was reached. The LOD threshold for QTL detection was calculated by permutation test with 1,000 iterations and a genome-wide significance level of 0.05.

### Anchoring to the Barley Physical Map and Identification of Candidate Genes

The new markers derived from Morex contigs, which flanked the resistance region after the QTL analysis, were employed for anchoring of genetic and physical maps following the instructions available at the FTP download page hosted at MIPS/IBIS (ftp://ftpmips.helmholtz-muenchen.de/plants/barley/public_data/anchoring). Once the putative regions conferring resistance to powdery mildew were delimited on the barley genomic sequence, the “high-confidence” (HC) and “low-confidence” (LC) genes on those regions were extracted according to the information available at ftp://ftpmips.gsf.de/plants/barley/public_data/anchoring/genes_to_physMap_08062012.tab and the EnsemblPlants website for barley http://plants.ensembl.org/Hordeum_vulgare. Annotation of HC genes was obtained from ftp://ftpmips.gsf.de/plants/barley/public_data/genes/barley_HighConf_genes_MIPS_23Mar12_HumReadDesc.txt. The putative function of LC genes was defined using gene ontology (GO) and PFAM protein motifs computed with InterproScan (http://www.ebi.ac.uk/Tools/pfa/iprscan).

Orthologous sequences of rice, Brachypodium and sorghum corresponding to HC candidate barley genes were identified by BlastP search against the rice annotation project database (RAP-DB; http://rapdb.dna.affrc.go.jp/tools/blast), the *Brachypodium distachyon* project at MIPS/IBIS (http://mips.helmholtz-muenchen.de/plant/brachypodium/) and the *Sorghum bicolor* database (http://www.phytozome.net/sorghum.php).

## Results

### Comparative Analysis to the Barley Genome Zipper

Markers QBS15 and GBM1060 on 7HS, markers QBS58 and QBS42 on 7HL and markers 11_1351 and 11_0509 on 6HL were designated as flanking markers for the QTL intervals according to previous works [20], [21].

**Figure 1:**
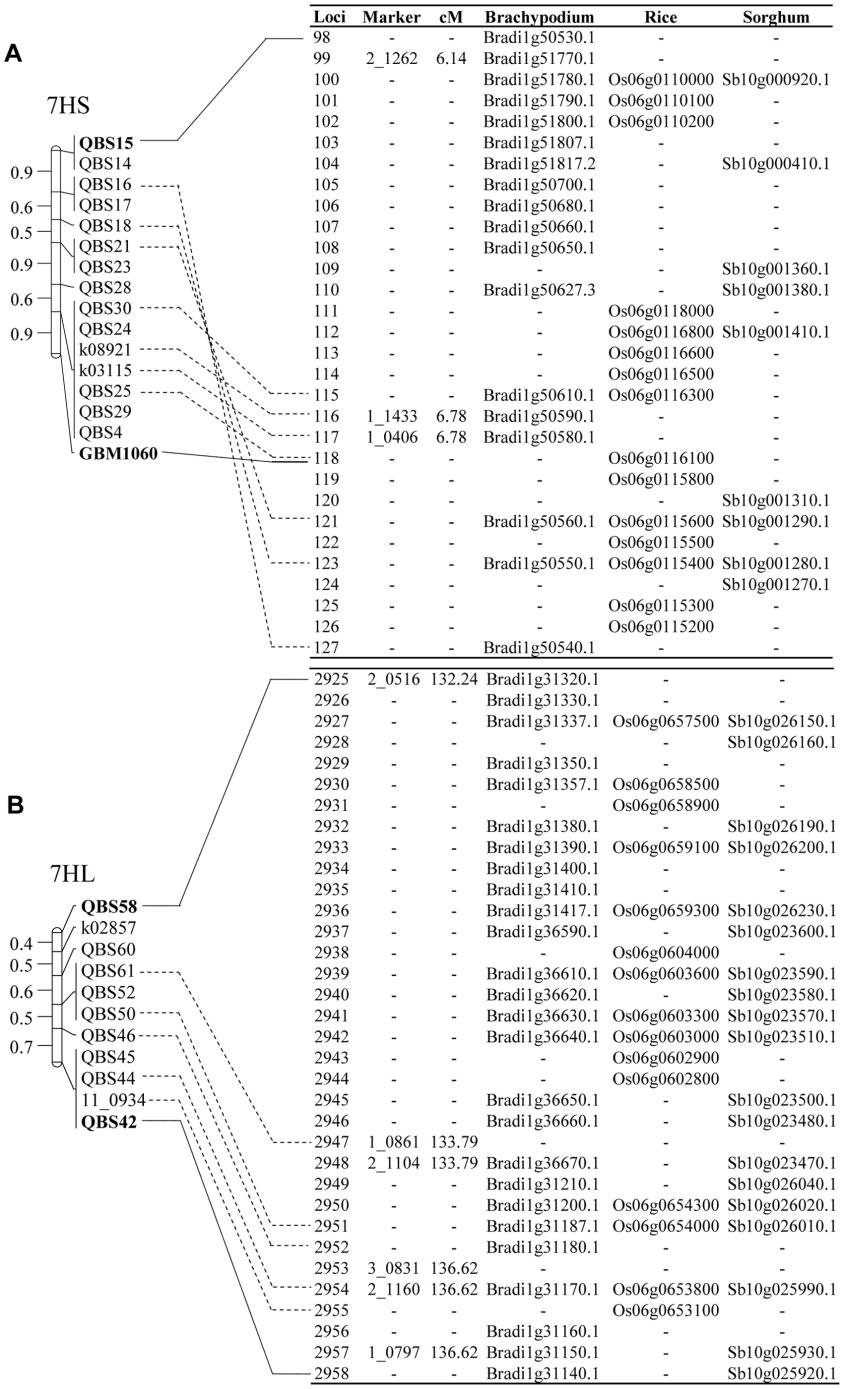
Anchoring of the QTL target intervals to the Genome Zipper of chromosome 7H. Comparison of chromosomes 7HS (A) and 7HL (B) genetic maps developed earlier [20] to the 7H Genome Zipper described by Mayer et al. [24]. For the sake of clarity, marker GBM1060 is only anchored to barley loci 118.

The data on the barley Genome Zipper held at the Barley Project hosted by the MIPS/IBIS was surveyed to find the barley unigenes, under the column “all non red. ESTs”, from which the flanking QBS markers were developed. This filtering of unigenes allowed the delimitation in the barley Genome Zipper of the regions corresponding to the two 7H intervals conferring resistance to powdery mildew in SBCC097. One region of 0.64 cM, holding 30 barley loci was delimited on the chromosome 7HS between markers QBS15 (unigene U35_32018) and GBM1060 (unigene U35_1176). The marker QBS15 only matched the Brachypodium gene Bradi1g50530.1, while GBM1060 matched genes Os06g0116100, Sb10g001310 and Bradi1g50590, confirming our previous results with the comparative genomic approach [20] (Fig. 1a). Most of QBS markers at the 7HS interval showed the same counterpart in the rice, sorghum and Brachypodium genomes, as predicted previously, although substantial reshuffling was noticed. Namely, the region from QBS16 to QBS21, which corresponds to barley positions from 121 to 127, appeared proximal to marker GBM1060 in the Genome Zipper, whereas it was located distal to this marker in our previous map (Fig. 1a). In preceding work, markers QBS23, QBS28 and QBS29 matched rice, sorghum and Brachypodium genes which are not represented in the Genome Zipper [20] (Fig. 1a). Surprisingly, the comparative analysis to barley predicted loci revealed the presence of two re-arrangements in rice, sorghum and Brachypodium genomes at the barley positions 99–104 (insertion) and 105–114 (inversion), which were not detected with the syntenic integration approach described earlier (Fig. 1a).

One region of ca. 4.30 cM, harboring 34 loci, was identified on the long arm of chromosome 7H, between flanking markers QBS58 (unigene U35_18765) and QBS42 (unigene U35_11617) (Fig. 1b). An unexpected good colinearity was observed between our genetic map and the linear order of barley genes predicted by the Genome Zipper, contradicting our previous results, which showed an inversion in the rice, sorghum and Brachypodium physical maps compared to the genetic region of barley flanked by QBS58 and 11_0115 [20]. Markers QBS60 and QBS61 detected rice, sorghum and Brachypodium genes which are not represented in the Genome Zipper. Once more, some re-organizations on the reference genomes, among predicted barley loci 2937–2948 were observed for the first time (Fig. 1b).

**Figure 2:**
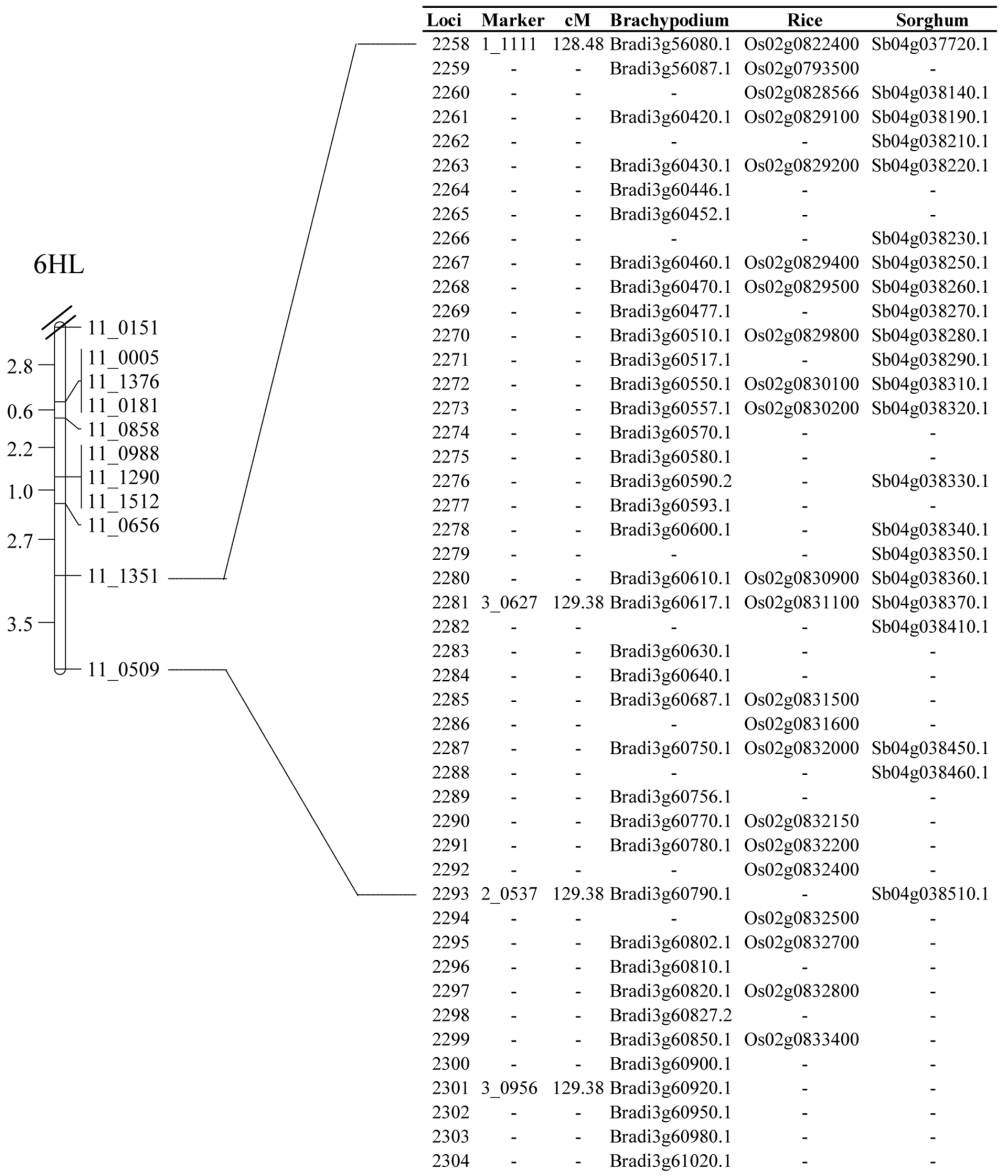
Anchoring of the QTL target intervals to the Genome Zipper of chromosome 6H. Comparison of chromosome 6HL genetic map developed earlier [20] to the 7H Genome Zipper described by Mayer et al. [24]. For the sake of clarity, only the telomeric part of chromosome 6H is represented.

The search for homology on chromosome 6H was directly based on BOPA markers. Flanking markers 11_1351 and 11_0509 were identified as markers 1_1111 and 2_0537, respectively, on the Genome Zipper, and one region of 0.9 cM, comprising 36 barley loci was defined (Fig. 2). This interval corresponded to a syntenic region on chromosome 2 (Os02) of rice (comprising 18 genes), chromosome 4 (Sb04) of sorghum (22 genes) and chromosome 3 (Bd03) of Brachypodium (28 genes). Due to the tricky position of the QTL at 6HL, which is located at the telomeric end of the chromosome, an additional region of 11 loci, proximal to marker 11_0509, was also selected for subsequent analysis (Fig. 2).

Fifteen, nine and twelve genes putatively located within the target intervals at 7HS, 7HL and 6HL, respectively, in the barley Genome Zipper were selected for further work (Table 1). In the case of the intriguing zipper loci 2953 (7HL), at which position no reference genes were available, the 454 read CUST_39488_PI390587928_13937_7HL was directly employed for marker development. The sequences of rice, Brachypodium and sorghum showing synteny with those barley loci were employed for ViroBlast search against the “assembly_WGSMorex” database. The Morex contig with the best hit at each locus was selected for further work (Table 1). E-values for the selected contigs after ViroBlast search ranged from 0.0 to 2E-42, with a length between 1661 to 19133 bp (Table 1). All contigs, except three on 7HS (contig_43731, contig_53679, contig_2552675) were assigned to the expected barley chromosome according to the information available at the “assembly_WGSMorex” database. Those contigs without assignation were blasted against the rice, sorghum and Brachypodium genomes, showing a great homology to the chromosomes Os06, Sb10 and Bd1 (data not shown), and therefore, they were also considered for further work on marker development. The 36 Morex sequences were employed in a second step of ViroBlast against the “Sorted Chromosomes” database in order to identify their homologous Betzes 454 reads. The number of reads identified per each contig at E-values smaller than e^−30^ ranged from 3 to 20, with an average of ∼14 reads per contig (Table 1). The alignment of the sequences from Morex contigs and Betzes reads allowed the identification of those regions with higher number of *in silico* SNPs, and permitted to focus the primer design on those intervals presumably containing the highest polymorphism rates.

**Table 1:** Morex contigs identified from the selected barley loci on chromosomes 7HS, 7HL and 6HL in Genome Zipper, and number of Betzes reads employed for the alignment to contigs and the detection of *in silico* SNPs.

Chrom.	Barley Loci	Morex Contig	E-value	Contig size (bp)	Betzes reads
7HS	99	86947	5.0E-67	5180	5
	100	39067	5.0E-149	19133	20
	101	39067	1.0E-176	19133	20
	103	56519	0.0	3100	14
	104	43731	0.0	6468	3
	105	45091	1.0E-122	9419	17
	106	56996	0.0	13259	19
	108	62161	1.0E-149	5735	16
	110	335030	0.0	8139	20
	111	53679	4.0E-62	7130	8
	112	275608	8.0E-85	2978	7
	113	2552675	4.0E-54	2651	4
	114	335030	0.0	8139	20
	120	6245	1.0E-128	7446	18
	126	45274	0.0	4892	16
7HL	2927	36988	2.0E-164	3995	20
	2933	102319	3.0E-136	6141	10
	2937	43456	0.0	18642	20
	2942	160008	9.0E-90	3156	16
	2946	135867	0.0	4214	16
	2948	1562518	0.0	5683	17
	2949	168471	4.0E-90	1661	5
	2950	7066	0.0	8199	20
	2953	CUST_39488	–	689	–
	2956	1561792	3.0E-32	2569	10
6HL	2261	98708	7.0E-177	11121	19
	2264	165059	0.0	3889	17
	2267	2549444	0.0	6855	16
	2270	138749	0.0	6971	13
	2273	160010	2.0E-101	5220	11
	2276	38804	0.0	6643	13
	2278	159682	3.0E-114	4182	7
	2281	50047	0.0	10166	5
	2285	66958	0.0	14049	14
	2287	1568412	3.0E-161	4360	12
	2291	57887	2.0E-42	6909	12
	2299	46523	4.0E-131	4512	13

Primers were designed for amplification and sequencing of promising regions - in the range of 1000 bp - on the selected barley contigs (Table S1). The contigs_86947 and 39067 on 7HS, contig_168471 on 7HL and contig_160010 on 6HL did not amplify any fragment with none of two primers pairs tested at different positions on the contig (Table S1). The marker QB_160008 on 7HL was monomorphic. The rest of markers developed from contigs resulted highly polymorphic between the parental lines, with an average number of 2 SNPs per 1 kb fragment (data not shown). The read CUST_39488_PI390587928_13937_7HL also discerned between SBCC097 and Plaisant. Thirty-one new markers (twelve at 7HS, eight at 7HL and eleven at 6HL) were genetically mapped in the lines selected from the two populations yielding thirty-one new loci. Five markers were genotyped based on the presence/absence of the PCR product, three were detected as a length polymorphism and the rest were genotyped as CAPS by using a restriction digestion assay (Table S1).

**Figure 3:**
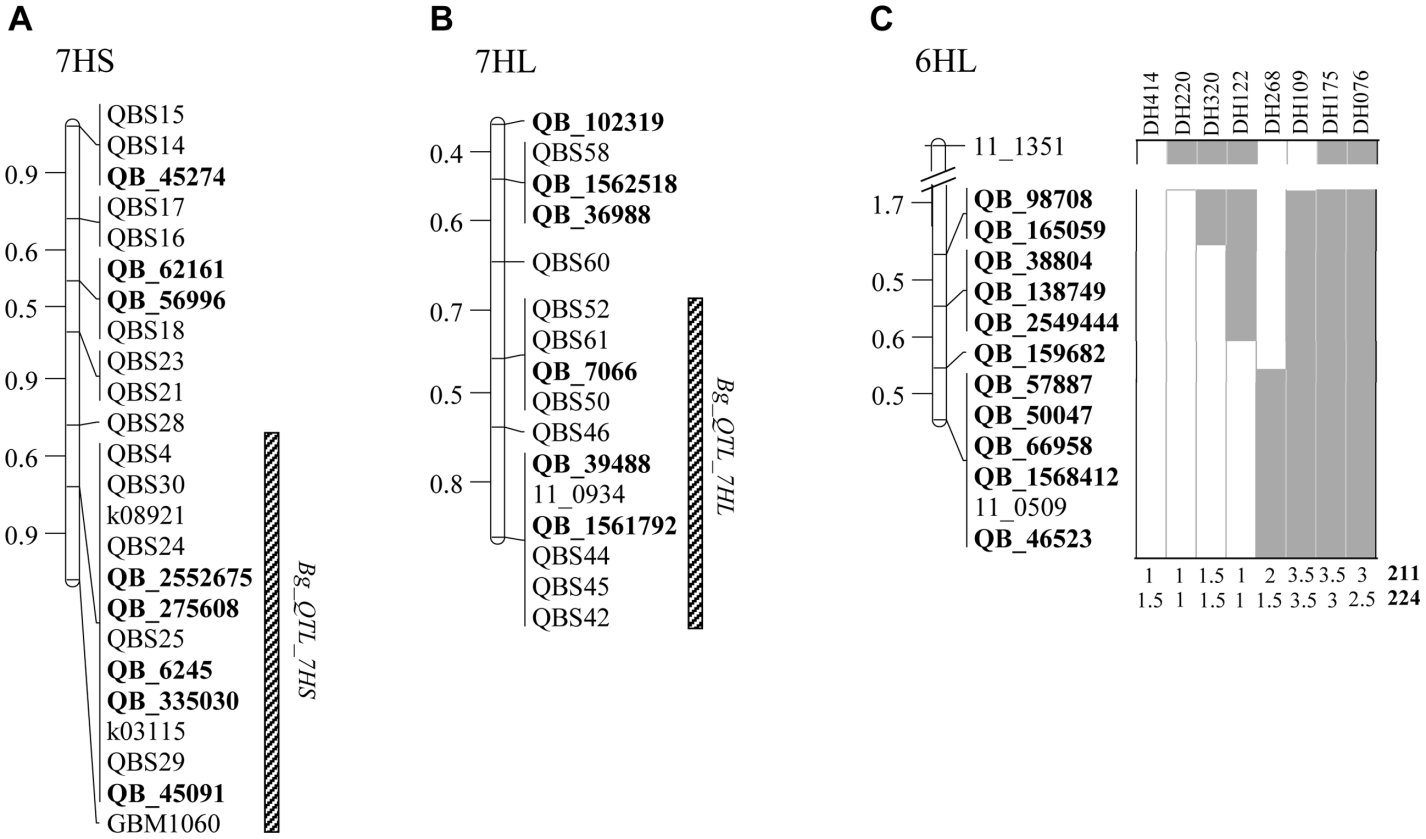
Genetic linkage maps after saturation with new contig-based markers. (A) chromosome 7HS, (B) 7HL and (C) 6HL. New markers are represented *in bold*. Scratched bars indicate the position of potential chromosomal regions conferring resistance to *B. graminis* in 7HS and 7HL. A diagram of the DH lines of the SBCC145×Beatrix population showing recombination on the regions harboring the QTL is presented for the chromosome 6HL. The disease score (ranging from 0 to 4) of each DH line is indicate for each isolate (211, 224).

Regarding the 7HS chromosome, the markers QB_39067, QB_56519, QB_43731 and QB_53679 mapped out of the selected interval (Fig. 3a). The other eight markers were located at the QTL region as expected, except for the marker QB_45274, which co-segregated with markers QBS15 and QBS14, at a different position of that predicted in the putative barley gene index of chromosome 7HS. Four out of eight markers from contigs and one read were mapped at the 7HL interval (Fig. 3b), one (QB_102319) was genetically mapped 0.4 cM distal to QBS58 and the other two (QB_135867 and QB_43456), which correspond to the predicted rearrangement at loci 2937–2948, were located out of the interval. The other markers mapped in good colinearity with their predicted positions in the Genome Zipper (Fig. 3b). All fourteen new markers positioned on chromosome 7H in the SBCC097×Plaisant population, co-segregated with other previously developed QBS markers. The constructed linkage map of 7HS and 7HL resulted in 7 and 5 groups, respectively, of non-identical co-segregating markers with a range of 0.5–0.9 cM and an average of 0.69 cM between groups (Fig. 3a,b). The contig-based markers did not provide a better delimitation of the 7H QTLs, but they allowed for an increment in marker density to 1 marker per 0.18 or 0.17 cM, within the target regions at 7HS and 7HL, respectively (Fig. 3a, b). All eleven markers developed from contigs on 6HL chromosome mapped as expected at the region spanning the QTL on SBCC145 (Fig. 3c). These new markers permitted to narrow down the chromosomal sections containing the QTLs. An examination of the three most informative lines (DH-122, DH-268 and DH-320) suggested the presence of the QTL co-segregating with the marker derived from contig_159682 (Fig. 3c).

### Anchoring to the Barley Physical Map and Identification of Candidate Genes

The information available for those Morex contigs bearing the new flanking markers on each QTL was used for anchoring the SBCC097×Plaisant and SBCC145×Beatrix genetic maps to the physical map of barley. A genomic region of 4 Mb was identified for the 7HS interval between markers QB_275608 and QB_45091 (Table 2). Morex contigs underlying those markers were anchored to FP contigs (FPC) and they were positioned according to the AC1 anchoring strategy described by IBSC [25]. Two regions of 3.7 Mb and 3.2 Mb covered the QTL intervals on 7HL and 6HL, between markers QB_1562518 and QB_1561792 and QB_138749 and QB_46523, respectively (Table 2). The Morex contigs anchored to FPC were positioned as described above, while those Morex contigs (morex_contig_1561792 for 7HL and morex_contig_46523 for 6HL) without sequence homology to any FPC were anchored according to the AC2 strategy [25]. All newly developed markers within the target intervals matched a FP contig or a Morex contig in the expected order, except for markers QB_7066 and QB_159682, whose Morex contigs were physically positioned elsewhere in the chromosome 7HL and 6HL, respectively.

**Table 2:** Anchoring of the genetic markers developed from Morex contigs to the physical map of barley via FP contigs (AC1 strategy) or Morex contigs (AC2 strategy) [25].

Chrom	Marker name	Morex_contig	FP contig	cM^1^	Bp^2^
7HS	QB_275608	morex_contig_275608	45784	8.286119	9055720
	QB_2552675	morex_contig_2552675	–	–	–
	QB_6245	morex_contig_6245	–	–	–
	QB_335030	morex_contig_335030	44369	12.747875	11229440
	QB_45091	morex_contig_45091	44313	12.747875	13155160
7HL	QB_1562518	morex_contig_1562518	1622	120.82153	574959480
	QB_36988	morex_contig_36988	–	–	–
	QB_7066	morex_contig_7066	–	–	–
	QB_1561792	morex_contig_1561792	–	124.57507	578735280
6HL	QB_138749	morex_contig_138749	8992	119.33428	535422080
	QB_2549444	morex_contig_2549444	48820	119.33428	537882240
	QB_159682	morex_contig_159682	–	–	–
	QB_57887	morex_contig_57887	–	123.79603	537882240
	QB_50047	morex_contig_50047	–	126.48725	537882240
	QB_66958	morex_contig_66958	7137	126.48725	538665920
	QB_1568412	morex_contig_1568412	–	–	–
	QB_46523	morex_contig_46523	–	126.6289	538665920

1 cM position according to IBSC [25], ^2 ^Bp position according to IBSC [25].

Only those Morex contigs bearing “high-confidence” or “low-confidence” genes, according to the definition established by IBSC [25], were considered for drawing a minimum tiling path at the three target genomic regions. The region putatively carrying the powdery mildew resistance on 7HS comprises 10 FPC and 98 Morex contigs (Table S2). Three FPC do not contain any Morex contig with an assigned gene. Fifty-nine morex contigs were anchored to the other seven FPCs (Table S2). This genomic region contains 99 LC and 53 HC genes. The region on 7HL displayed 10 FPC, 84 morex contigs and 122 genes (81 LC and 41 HC) (Table S2). Thirty-six Morex contigs were anchored to seven FPC and the additional three FPC did not have any gene assigned (Table S2). Regarding the genomic interval on 6HL, the physical region covered 6 FPC and 66 Morex contigs (Table S2). Forty-one of these contigs were anchored to FPC. In total, 87 genes (47 LC and 40 HC) were assigned to this region (Table S2).

Among the genes annotated from the Morex contigs on the 7HS, 7HL and 6HL regions, 10, 7 and 10 HC genes, respectively, showed a functional annotation that hints to an involvement in the disease resistance mechanisms. They might be considered as candidates for the resistances described at these genomic regions (Table 3, Table S2). In total, five genes were annotated as Nucleotide Binding (NB)-Leucine Rich Repeats (LRR) proteins (PFAM: PF00931), four were identified as belonging to the serine/threonine protein kinase (S/TPK) family (PF00069), and two genes contained domains that could be involved in the recognition of the pathogen (Table 3). Additional candidate genes encoded proteins that could be involved in other mechanisms of plant defense or signal transduction more than in the perception of pathogen effectors (Table 3). BlastP search for orthologous genes in the three grasseś reference genomes showed low levels of microsynteny for the target regions. In total, six (22.2%), eight (29.6%) and eleven (40.7%) proteins predicted from the most promising HC genes matched their corresponding counterpart on the expected chromosome of rice, sorghum and Brachypodium, respectively (Table 3).

**Table 3:** Barley “high-confidence” candidate genes identified at the target QTL intervals and protein orthology to rice, Brachypodium and sorghum genes.

Chrom	Barley gene	FP contig	Morex_contig	Annotation	Protein lenght	PFAM	Rice	Brachypodium	Sorghum
7HS	AK251676.1	–	morex_contig_6245	Protein kinase superfamily protein LENGTH = 464	483	PF00069	Os02t0700600-01	Bradi1g50590.1	Sb10g001310.1
	MLOC_5217.3	–	morex_contig_136027	Disease resistance protein (CC-NBS-LRR)	1035	PF00931	Os11t0704100-00	Bradi1g00290.1	Sb05g004310.1
	MLOC_70200.1	contig_41890	morex_contig_57570	Leucine-rich repeat receptor kinase-like protein	884	PF00560	Os11t0208900-01	Bradi4g10987.1	Sb05g027140.1
	MLOC_63994.1	contig_44369	morex_contig_48252	F-box domain containing protein	251	–	Os06t0113600-01	Bradi1g50426.1	Sb05g001110.1
	MLOC_72805.2	contig_44369	morex_contig_6241	NBS-LRR disease resistance protein, putative, expressed	914	PF00931	Os10t0358200-00	Bradi5g01167.1	Sb02g027790.1
	MLOC_7548.1	contig_44369	morex_contig_139505	Protein kinase-3	427	PF00069	Os11t0608300-01	Bradi4g09457.1	Sb09g000630.1
	MLOC_69817.1	contig_44369	morex_contig_64353	Superoxide dismutase	393	PF02777	Os06t0115400-01	Bradi1g50550.1	Sb10g001280.1
	MLOC_16184.1	–	morex_contig_1572657	Peroxidase 1	324	PF00141	Os07t0157000-00	Bradi2g11300.1	Sb09g002760.1
	AK250430.1	–	morex_contig_43906	GDSL esterase/lipase	382	PF00657	Os01t0223200-01	Bradi2g07320.1	Sb03g001320.1
	MLOC_75548.1	–	morex_contig_68187	Protein kinase domain containing protein, expressed	100	–	–	–	–
7HL	MLOC_60881.1	contig_591	morex_contig_44875	WD-repeat protein 57	346	PF00400	Os06t0653800-01	Bradi1g31170.1	Sb10g025990,1
	MLOC_2395.1	contig_591	morex_contig_121333	Leaf rust resistance protein Lr10	464	–	Os11t0249000-01	Bradi2g39537.1	Sb07g020160.1
	MLOC_5842.1	contig_3887	morex_contig_136766	GDSL esterase/lipase	370	PF00657	Os01t0651000-01	Bradi2g45230.1	Sb03g029600.1
	MLOC_39511.4	–	morex_contig_2553727	Phospholipase D4	413	PF08371	Os06t0649900-01	Bradi1g30930.2	Sb10g025660.1
	MLOC_11419.3	contig_6970	morex_contig_1561039	Protein kinase superfamily protein LENGTH = 579	606	PF00069	Os08t0249100-01	Bradi3g19020.1	Sb07g008540.1
	MLOC_62970.1	–	morex_contig_47144	F-box domain containing protein, expressed	410	PF00646	Os12t0128000-01	Bradi1g31010.1	Sb01g037880.1
	MLOC_39346.1	–	morex_contig_2553094	GDSL esterase/lipase	359	PF00657	Os01t0651000-01	Bradi2g45230.1	Sb03g029600.1
6HL	AK250145.1	–	morex_contig_61285	Leucine-rich repeat-containing protein 40	586	PF00560	Os02t0826600-01	Bradi3g56757.1	Sb04g038010.1
	MLOC_16806.1	contig_43670	morex_contig_1574545	NBS-LRR resistance protein	192	PF00931	–	–	–
	MLOC_71605.1	contig_43670	morex_contig_6016	NBS-LRR class disease resistance protein	555	PF00931	Os08t0539400-01	Bradi2g39517.1	Sb02g006060.1
	MLOC_43786.1	contig_43670	morex_contig_272682	Disease resistance protein	591	PF00931	Os08t0539400-01	Bradi2g39517.1	Sb05g022785.1
	MLOC_65331.1	–	morex_contig_50051	70 kDa heat shock protein	608	PF00012	Os03t0276500-01	Bradi1g66590.1	Sb01g039530.1
	MLOC_21622.1	–	morex_contig_159682	Receptor-kinase, putative	242	PF00069	Os04t0649700-02	Bradi3g60600.1	Sb04g038340.1
	MLOC_14187.1	–	morex_contig_1567476	F-box domain containing protein	381	–	Os06t0111200-01	Bradi1g50370.1	Sb10g001090.1
	AK373189	–	morex_contig_50047	Zinc finger CCCH domain-containing protein 19	522	–	Os02t0831100-01	Bradi3g60617.1	Sb04g038370.1
	MLOC_75709.2	contig_7137	morex_contig_6865	Glucan synthase-like 2	1405	PF02364	Os01t0754200-03	Bradi3g60790.2	Sb04g038510.1
	MLOC_44276.3	contig_7137	morex_contig_274445	NBS-LRR disease resistance protein homologue	949	PF00931	Os04t0621500-00	Bradi5g22187.1	Sb06g028930.1

Analysis of GO/PFAM terms on the protein sequences derived from LC genes revealed that 11 (7HS), 3 (7HL) and 6 (6HL) genes might be involved in the disease resistance, exhibiting mainly the terms GO:0005524 (ATP-binding), GO:0005515 (protein-binding), GO:0004672 (protein kinase activity), PF00069 (protein kinase domain), PF08263 (Leucine rich repeat N-terminal domain) and PF00931 (NB-ARC domain) (Table S2).

## Discussion

Positioning of disease resistance QTL on the physical map of barley constitutes an essential step towards the map-based gene cloning, but it also paves the way towards the suitable exploitation of these resources in breeding programs, through the development of tightly linked molecular markers. In barley, such steps were typically hampered by the large genome size of the crop and its highly repetitive nature [30]. Advances in barley genomics have abounded over the past decade greatly increasing the opportunities for interrogating the molecular mechanisms underlying the formation of interesting traits [31]–[36]. Among these, two recent milestones stand out as the main contributions to facilitate the access and full exploitation of the barley genome sequence. First, the construction of a genome-wide putative linear gene index of barley (Genome Zipper) based on flow sorted chromosomes and shotgun sequencing [24] and, more recently, the publication of the first draft of the physical, genetic and functional sequence of the barley genome [25]. The combined use of these resources allows reaching enhanced resolution of an extremely complex genome, making full use of the resolution available in classical biparental populations used for QTL search. We report here an example of this use to dissect to new depths the chromosomal regions conferring resistance to *B. graminis* in two Spanish barleys and as a conduit to identify candidate genes at the target intervals.

### Comparative Analysis to the Barley Genome Zipper

First, we took advantage of the previously established barley Genome Zippers of chromosomes 7H and 6H, constructed by integrating next generation sequencing information of barley with their synteny to reference grass genomes on individually purified barley chromosomes [24]. Comparison of our previous genetic maps with the virtual high-density gene maps of barley allowed the identification of three regions with homology to the expected chromosomes on rice, sorghum and Brachypodium according to other reports [37], [38]. Surprisingly, some rearrangements in the chromosome 7H, which were not detected in our previous work, were now identified within the regions spanning the QTLs. The information on the barley Genome Zipper at these loci was exploited for further development of tightly linked markers. To this end, barley contigs derived from an Illumina whole genome shotgun approach on cv. Morex were identified and employed for *in silico* detection of SNPs based on their alignment with 454 reads from cv. Betzes. Few contigs on 7HS, 7HL and 6HL could not be amplified on parental lines with any primer set located at different positions. Among them, contigs_86947 and 168471 gave an amplicon when tested in cv. Morex, suggesting variations in the genome sequence of the Spanish lines, something not unexpected considering that we are comparing old landraces with the sequence of modern cultivars. For the other contigs and reads, primer design on *in silico* selected regions was highly successful. Of the thirty-two primers pairs tested for polymorphism among parental lines, thirty-one (96.9%) generated useful amplicons. This polymorphism rate was much higher than that found in our and other previous works based on barley ESTs or unigenes [20], [39], [40]. Twenty-five percent of markers were genotyped either as a length polymorphism or based on presence/absence of the amplicon at one of the parental lines. Most commonly, the different fragment size or the absence of the PCR product was associated with the Spanish lines, which seems to support above data on the distinctive performance of barley landraces in comparison to modern cultivars.

The genetic maps obtained with the new markers were not in complete accordance with the putative linear gene order described in the Genome Zipper. Thus, the insertion postulated in the 7H virtually ordered gene inventory, between barley loci 99–104 (7HS) and loci 2937–2948 (7HL), were not confirmed in our results, which on the contrary, suggest an upstream location for the homologous genes in rice, sorghum and Brachypodium. Such absence of synteny was also observed for the inverted regions on chromosomes 7HS and 7HL, in which new markers were not genetically mapped according to the gene order expected from the Genome Zipper. Changes in markers order could be attributed to the fact that some of the barley loci anchored at those positions along the Genome Zipper of chromosome 7H are supported only by the order of their counterpart in one or two reference genomes [24]. This could explain some misinterpretation in the gene order when constructing the virtual barley model. At this medium resolution level of synteny, it is expected that the accuracy of the one-to-one relationship between orthologous will vary depending of the density of reference genomes and the ancestral rearrangements affecting few linked or unlinked genes under selection pressure [41], [42]. Several reports also demonstrated that colinearity is commonly less conserved at the telomeric regions of the chromosomes [43], [44], which is the case for 7HS and 6HL. Beyond this, a good performance was observed for the Genome Zipper, allowing the positioning of eight and five new markers at the 7HS and 7HL intervals, respectively. Regarding the 6HL region, a high level of colinearity was found between our new genetic map and the barley Genome Zipper, with all new markers developed from contigs ordered according to the position defined by the barley virtual map. Similar results have been found for chromosomes 1H, 2H and 4H (D. Perovic, unpublished data). These data suggest that the Genome Zipper should be retained as an extremely powerful resource for fine mapping, chromosome dissection and physical map anchoring, provided that such approach will also meet some limitations depending on the features of the target region.

### Anchoring to the Barley Physical Map and Identification of Candidate Genes

The addition of new markers to the 7H linkage map did not shed much more light to resolve the resistance regions, due to the resolution of the current mapping population. However, they will be very useful for a more precise screening for recombinants of large F2 populations and further positional isolation, allowing increase in marker density at the high-resolution mapping population from 1 marker/0.54 cM to 1 marker/0.27 cM in the case of 7HL (unpublished data). Regarding the QTL on 6HL, the addition of new markers narrowed down the resistance regions to smaller intervals, i.e. from 3.3 cM to 1.1 cM (1 marker/0.1 cM), and pointed out to QB_159682 as the most likely marker co-segregating with the QTL (based on the recombination events displayed by three DH lines). The generation of these saturated genetic maps is useful to exploit fully the potential of physical maps for map-based cloning strategies, since the sequenced contigs should be anchored at a density as high as possible with molecular markers [45]. Dense genetic maps are also valuable for the breeding community, to perform precise introgression of the novel resistances in elite cultivars via marker-assisted selection approaches.

These new markers developed from Morex contigs provided the framework for anchoring of genetic and physical maps, figuring out the outline of the barley genome at these regions conferring resistance in SBCC097 and SBCC145. The outermost flanking markers of QTLs on 7HS, 7HL and 6HL defined a physical area of 4 Mb, 3.7 Mb and 3.2 Mb, respectively. The accurate relationship of physical to genetic distance was hard to predict for the target loci due to co-segregation of markers. According to the map of Künzel et al. [46], the recombination rate at the distal end of chromosome 7HS corresponded to 1.3 Mb/cM. Our estimation, considering the block of co-segregating markers, was the ∼4 Mb/cM, which is three times larger than expected. This may be partially explained by the presence of two important gaps of 1.4 (between 9824520 and 11229440 bp) and 1.2 Mb (from 11933760 to 13155160 bp) in the barley genomic sequence predicted for this region. Correcting for this fact, the obtained ratio would be ∼1.4 Mb/cM, which is closer to that reported by Künzel et al. [46]. The recombination rate of the regions flanking the 7HL co-segregating area was of ∼1.5 Mb/cM, which is in accordance with Künzel et al. [46], who proposed rates between 1.8 and 3.4 Mb/cM. The estimated physical to genetic ratio for the 6HL was of 2.9 Mb/cM, which is within the predictions of Künzel et al. [46] (≤2.7 – ∼2.3 Mb/cM) for the distal end of chromosome 6HL. We expect that, the screening of high resolution populations of several thousands of individuals, which is in process, will allow narrowing down and delimiting more accurately the physical position of the each target locus to the size of one or few BAC clones.

Physical mapping of the 7HS, 7HL and 6HL regions identified 10, 10, 6 FP contigs and 39, 48, 25 non-anchored Morex contigs, which harbor a total of 152, 122 and 87 genes (both HC and LC), respectively. Twenty-six out of 134 (19.4%) HC genes were annotated as “unknown protein” or “Protein of unknown function”. In total, 21, 10 and 16 genes in 7HS, 7HL and 6HL, respectively, could be interpreted as potential candidates to explain the resistance to powdery mildew, as they encode proteins of related functions with respect to the pathogen defense-related processes. The majority of these were annotated as belonging to the NBS-LRR class or protein kinase family, which collectively represents the two most important groups of resistance genes cloned and characterized to date [47], [48]. Up to 5 protein kinases and 14 disease resistance proteins were identified on the short arm of chromosome 7H. This region has been previously described as a “hot spot” of recombination harboring many agronomical important traits, including several NBS-LRR and serine/threonine protein kinase (S/TPK) resistance genes [49]–[51]. Likewise, but more unexpected, were the 12 candidate genes detected at the target interval on chromosome 6HL, which also exhibited the structure of disease resistance proteins. Both results are in agreement with data from the IBSC [25] who reported up to 191 NBS-LRR type genes, which tended to cluster in gene families towards the distal ends of barley chromosomes.

The largest class of plant resistance genes encodes a NBS-LRR class of proteins [47]. The carboxy-terminal LRR domains are found in diverse proteins and function in the recognition of pathogen effectors as sites of protein–protein interaction [52]. The nucleotide-binding site (also termed as NB-ARC) is part of a larger domain with homology to some eukaryotic cell death effectors and it seems to play a role in the subsequent signaling events that trigger the resistance, through the hydrolysis of ATP [53], [54]. S/TPKs are another important group of resistance genes which may act directly conferring resistance to the pathogen or indirectly through its cooperation with a NBS-LRR gene, as happens with the tomato *Pto* gene [55], [56]. This collaboration of different protein domains to provide resistance to plant pathogenic organisms could explain their grouped positions at the distal regions of barley chromosomes, as they may sometimes work together for the resistance response to occur. One additional gene was annotated as a “Leucine-rich repeat receptor kinase-like protein”, which constitutes a third class of relatively few members that possesses the LRR and PK domains within the same transcript [57]. Another HC gene was annotated as a “WD-repeat protein 57″, which does not display the typical structure of a resistance protein but it maintains a domain (WD40) that could be involved in protein-protein interactions, in a similar way as the LRR domains [58]. Apart from these, other annotated genes were also judged as putative candidates based on the predicted function of the translated protein. Thus, CCCH-type zinc finger proteins, glucan synthase-like proteins, superoxide dismutase, peroxidase, GDSL esterase, etc, seem to play important roles in imparting host resistance through some function in the signaling networks triggering the multilayered mechanisms involved in the defense response [59]–[62].

Orthologous rice, Brachypodium and sorghum genes were identified for the most promising barley candidate genes at the protein level. Only ten of them (40%) lay in at least one of the syntenous regions described previously [37]. The number of conserved syntenic loci was similar in comparison with rice and sorghum (22.2 and 29.6%, respectively) but was higher with Brachypodium (40.7%), confirming a closer relationship and a better conservation of genetic material between this grass and the Triticeae [40], [63]. The lack of large microsynteny suggests that the regions conferring resistance to powdery mildew in Spanish barleys likely underwent some rearrangements compared to the three reference genomes. These results provide additional clues that explain the frequent lack of success of comparative genomics approaches for gene isolation in the Triticeae and support previous reports that suggested unique features in the barley genome [64]. Thus, the barley genes *ROR2*, *rym4/5* and *Ppd-H1* are all present within the syntenic positions of the rice genome [65]–[67]. However, the orthologs of the barley genes *Vrs1*, *Rpg1* or *Rdg2a* are either within non-syntenic positions or absent in the rice genome [41], [49], [68]. Such limited success of synteny-based strategies, even when integrating more than one reference genome, is frequently observed at disease resistance loci, which are particularly unstable and frequently subjugated to tandem or segmental duplications of the entire chromosomal regions where they are allocated [69], [70].

One marker on 7HS and two markers on 6HL were directly associated with candidate genes. Additionally, all the Morex contigs anchored to FP contigs could be used to identify BAC clones from the Morex BAC libraries, according to the data available at IBSC [25]. This information together with further analysis of high-resolution mapping populations, which were separately constructed for the analysis of each QTL independently, will serve to construct a more accurate and reliable minimum tiling path containing the regions that confer resistances to powdery mildew in Spanish barley landraces. As far as we know, the current report places among the earliest efforts to put into practice the recently developed barley genomic resources to deal with old breeding dilemmas, such as accurate identification and exploitation of novel disease resistances.

## Supporting Information

Table S1 **Contig-based markers developed and evaluated for the marker enrichment of the chromosome 7HS, 7HL and 6HL regions harboring the QTLs for resistance to powdery mildew.**
(XLS)

Table S2 **Anchoring of genetic and physical maps of barley.** Location on the barley physical map of the regions putatively carrying the powdery mildew resistance on 7HS, 7HL and 6HL. The HC, LC genes, Morex contigs and FP contigs include on each interval are represented. Annotation makes reference to HC genes, while GO and PFAM Terms are shown only for LC genes.
(XLS)
